# Editorial: Spermatogenesis: from stem cells to spermatozoa

**DOI:** 10.3389/fendo.2023.1224313

**Published:** 2023-05-25

**Authors:** Gerhard Haidl, Ludovic Dumont

**Affiliations:** ^1^ Department of Dermatology, University of Bonn, Bonn, Germany; ^2^ Univ Rouen Normandie, French National Institute for Health and Medical Research (INSERM), Neuroendocrine Endocrine and Germinal Differentiation and Communication (NorDiC) UMR 1239 – Team Adrenal and Gonadal Pathophysiology (AGoPath), Rouen, France

**Keywords:** male infertility, spermatogenesis, stem cells, sperm morphology, germ cell culture

Due to the undoubtedly rather big success of artificial reproductive techniques attention in clinical reproductive medicine is mainly laid on progress in this field in order to achieve even better success rates. With regard to male infertility basic research has yielded also remarkable new results in terms of better understanding causes of male fertility disturbances where the majority of research efforts comes from genetic studies. Many new genes being involved in spermatogenesis and their potential relation to specific spermatogenic disturbances, in particular the meiotic division process, have been identified. This is, of course, an important development, which does, on the other hand, not really help the affected couple in their aim to become finally parents, because they only are informed why it does not work. In addition, there seems to be a gap between basic research in Andrology and its clinical application. What one has to keep in mind is the more or less unique structural principle of the human seminiferous tubules. Whereas in the majority of mammalians major areas of the testis are covered by the association of identical cells in a so-called stage with only minor differences of differentiation and hormonal regulation by testosterone and FSH, in the human these areas confine to a much smaller space. Therefore, areas that require a maximum of testosterone and a minimum of FSH are located directly next to those needing just the contrary relation of hormones. This leads inevitably to irritations of the development of germinal cells by minimal damaging factors. This is one of the reasons why we observe a highly varied picture of sperm morphology with only a very low percentage of normally shaped spermatozoa. Obviously, this still is enough to sustain normal reproductive capacity. Knowledge of these vulnerable basic mechanisms and regulatory factors including testicular anatomy, physiology, endocrinology and genetics ranging to potential environmental influences to this highly complicated process of spermatogenesis is prerequisite in order to understand male fertility and its disturbances and to look for reasonable solutions to overcome the problem.

Approaches to management of disturbed male fertility comprise avoidance of potential harmful external influences, medical treatment or, if such measures are not applicable, cryopreservation of spermatozoa or testicular tissue before fertility damaging interventions. Most challenging tasks are new techniques such as stem cell culturing and germ cell transplantation.

All these aspects are addressed in this Research Topic.

Horvath-Pereira et al. report on the current state and future perspectives in using biomaterials for testicular bioengineering. The most promising approaches to preserve male fertility comprises testicular cryopreservation, and, in cases where this is not applicable germ cell transplantation and testicular grafts. In this highly forward-looking contribution the application of bioengineering with various types of biomaterials including extracellular matrix is highlighted, which sounds very promising, not least as these techniques can replace the requirement for experimental animals, a big progress.

As pointed out earlier the first steps of spermatogenesis are the most crucial ones for further development of the germ cells. One of the most challenging issues in the future represents suitable cryopreservation techniques of prepubertal testicular tissue for boys who have to undergo fertility damaging treatments, mostly because of cancer. In their elegant animal study, Dumont et al. have demonstrated that testicular tissue freezing has only minor impact on gene expression. In addition, they pointed out that a disorganized steroidogenic pathway and probably inflammation could explain differences between *in vivo* and physiological controls. Moreover, ways to further optimization of organotypic culture procedures are illustrated.

Although gonadotrophins have been used since decades for treatment of male hypogonadism and infertility there are still some critical questions to be answered in order to optimize the management of male reproductive health. Bhattacharya et al. emphasize the necessity to generate translational progress in terms of clinical success in order to improve male infertility and to develop reversal male contraception methods. For this purpose identification of further FSH- and LH(T) responsive genes is required.

The highly relevant contribution by Lai et al. highlights the harmful influence of thoracic X-ray radiation on spermatogenesis in mice with all the consequences of increased pro-inflammatory cytokines. A problem that could be minimized if one were aware of it.

So, this plethora of information providing new and promising perspectives for male fertility problems should be attractive for all being active in reproductive medicine.

## Author contributions

The author confirms being the sole contributor of this work and has approved it for publication.

