# Corrigendum: Editorial: Spermatogenesis: from stem cells to spermatozoa

**DOI:** 10.3389/fendo.2023.1298519

**Published:** 2023-10-10

**Authors:** Gerhard Haidl, Ludovic Dumont

**Affiliations:** ^1^ Department of Dermatology, University of Bonn, Bonn, Germany; ^2^ Univ Rouen Normandie, French National Institute for Health and Medical Research (INSERM), Neuroendocrine Endocrine and Germinal Differentiation and Communication (NorDiC) UMR 1239 – Team Adrenal and Gonadal Pathophysiology (AGoPath), Rouen, France

**Keywords:** male infertility, spermatogenesis, stem cells, sperm morphology, germ cell culture

**Incorrect Affiliation**

In the published article, there was an error in affiliation 1. Instead of “Department of Dermatology, University of Bonn, Bonn, Germany” for Gerhard Haidl, it should be “^1^ Department of Dermatology, University of Bonn, Bonn, Germany”.

The authors apologize for this error and state that this does not change the scientific conclusions of the article in any way. The original article has been updated.

**Additional Affiliation(s)**

In the published article, there was an error regarding the affiliation for Ludovic Dumont. As well as having affiliation “none”, they should also have “^2^ Univ Rouen Normandie, INSERM, NORDIC UMR 1239 – Team Adrenal and Gonadal Pathophysiology (AGoPath), Rouen, France” for Ludovic Dumont.

The authors apologize for this error and state that this does not change the scientific conclusions of the article in any way. The original article has been updated.

**Error in Author List**

In the published article, there was an error in the author list, and author Ludovic Dumont was erroneously excluded. The corrected author list appears below.

Gerhard Haidl, Ludovic Dumont

The authors apologize for this error and state that this does not change the scientific conclusions of the article in any way. The original article has been updated.

**Text Correction**

In the published article, there was an error. Thanks are due to Dr Ludovic Dumont, who edited all 3 articles accepted in this Special Volume and was the first author of another article published in it. His name should therefore appear in this Editorial as an author (and not in the Acknowledgments’ Part. In addition, please modify in accordance the “Edited and Reviewed by” and “Citation” Sections.

• A correction has been made to **Acknowledgments**. This sentence previously stated:

“Thanks to Dr. Dumont for fine cooperation and assistance.”

The corrected sentence appears below:

““

• A correction has been made to **Edited and Reviewed by**. This sentence previously stated:

“Richard Ivell, University of Nottingham, United Kingdom.”

The corrected sentence appears below:

“Richard Ivell, University of Nottingham, United Kingdom

Ludovic Dumont, University of Rouen Normandy, France”

• A correction has been made to **Citation**. This sentence previously stated:

“Haidl G (2023) Editorial: Spermatogenesis: from stem cells tospermatozoa. Front. Endocrinol. 14:1224313. doi: 10.3389/fendo.2023.1224313.”

The corrected sentence appears below:

“Haidl G and Dumont L (2023) Editorial: Spermatogenesis: from stem cells to spermatozoa. Front. Endocrinol. 14:1224313. doi: 10.3389/fendo.2023.1224313”

The authors apologize for this error and state that this does not change the scientific conclusions of the article in any way. The original article has been updated.

